# Molecular epidemiological analysis of *Vibrio parahaemolyticus* in foodborne diseases in Liaoning province, 2023–2024

**DOI:** 10.3389/fpubh.2026.1780398

**Published:** 2026-03-31

**Authors:** Weijie Wang, Xiangyun Liu, Lianzheng Yu, Mingyan Zhang, Tongzhu Wei, Xue Li, Jinghong Ma, Taoran Zhao, Yue Zheng, Qingyuan Wang, Yan Wang, Wenli Diao

**Affiliations:** 1 Liaoning Provincial Center for Disease Control and Prevention, Shenyang, Liaoning, China; 2 China Medical University, Shenyang, Liaoning, China

**Keywords:** foodborne illness, MLST, molecular epidemiology, *Vibrio parahaemolyticus*, whole-genome sequencing

## Abstract

**Introduction:**

Vibrio parahaemolyticus (VP) is a major pathogen causing foodborne diseases, and Liaoning Province, a coastal region with high seafood consumption and production, faces a notable risk of VP-associated foodborne infections. Clarifying the molecular epidemiological characteristics of VP in this region is critical for targeted prevention and control of such diseases. This study aimed to investigate the molecular epidemiological features, virulence gene profiles, and antibiotic resistance gene distribution of VP isolated from foodborne disease cases in Liaoning Province during 2023-2024, providing precise data support for VP prevention and control.

**Methods:**

Whole-genome sequencing (WGS) was performed on 261 clinical isolates of *Vibrio parahaemolyticus* collected in Liaoning province from 2023 to 2024. Multilocus sequence typing (MLST), virulence gene identification, and antibiotic resistance gene detection were conducted and combined with epidemiological surveillance data to analyze the association between genetic and epidemiological characteristics.

**Results:**

A total of 10,413 foodborne disease cases were monitored during 2023–2024, with 261 isolates of *Vibrio parahaemolyticus* identifed (detection rate: 2.51%). The positive rate in 2024 (3.59%) was significantly higher than that in 2023 (1.27%, *P* < 0.05). *Vibrio parahaemolyticus* infections showed obvious seasonal characteristics, with the majority of cases occurring in July–September, with a peak in August, and consistent seasonal epidemic dynamics were observed between 2023 and 2024. Aquatic animals and their products (including fish, shellfish, crustaceans, and their fresh/processed products) were the most common exposure food category (52.11%). Restaurants (37.55%) and homes (34.87%) were the main eating venues for infected cases. Among antibiotic resistance genes, *tet* (34) and *bla*CARB-22 had the highest carriage rates (100% and 94.64%, respectively). Among the 71 detected virulence genes, 61 were core virulence genes shared by all isolates, and *flg*B had the highest deletion rate (95.79%). MLST analysis identified 11 sequence type based on typing results, with ST3 as the dominant epidemic strain (94.25%).

**Conclusion:**

*Vibrio parahaemolyticus* infections in Liaoning province have clear and stable seasonal characteristics during 2023–2024, with aquatic animals and their products as the main exposure source. ST3 is the dominant epidemic strain and is associated with the carriage of *tet* (34), *bla*CARB-22, and a variety of virulence genes based on genomic detection. The resistance gene profile of *Vibrio parahaemolyticus* isolates shows regional heterogeneity in Liaoning Province. Strengthening seafood safety supervision and continuous monitoring of the ST3 strain, as well as tracking of multidrug-resistant isolates, are of great significance for the scientific prevention and control of *Vibrio parahaemolyticus* foodborne diseases.

## 1 Introduction

Foodborne diseases refer to acute or chronic symptoms caused by bacteria, viruses, or other harmful substances entering the human body through ingestion, mainly manifested as gastrointestinal symptoms such as nausea, vomiting, diarrhea, and abdominal discomfort. It is estimated that 10.74 million foodborne illness cases occur in China each year (1). *Vibrio parahaemolyticus* is widely distributed in seawater, marine sediments and various shellfish, and eating undercooked or contaminated seafood is the main route of human infection (2–4). This bacterium can also cause wound infections that progress to systemic sepsis in immunocompromised individuals (5, 6), and its incidence in inland locations has increased with the expansion of seafood distribution networks. In China, *Vibrio parahaemolyticus* has been the primary cause of sporadic cases for consecutive years, with a higher incidence of foodborne diseases than *Shigella* and *Salmonella* (7, 8).

As a coastal province, Liaoning produces and consumes large quantities of seafood, therefore clarifying the epidemiological and molecular characteristics of *Vibrio parahaemolyticus*-induced foodborne diseases in this region is crucial for food safety assurance, public health protection, and evidence-based prevention and control strategies. WGS technology has been widely applied in microbial research, enabling rapid characterization of bacterial population genotypes through bioinformatics analysis of sequencing data (9–11). This study integrated WGS-based MLST, virulence gene identification and antibiotic resistance gene detection, and combined these with epidemiological data to describe the association between genetic characteristics of *Vibrio parahaemolyticus* strains isolated from foodborne disease cases in Liaoning province during 2023–2024. We systematically explored the correlations (descriptive, non-causal) between genetic attributes, epidemiological features, and clinical manifestations of the strains, in order to clarify the epidemic trends and genetic characteristics of *Vibrio parahaemolyticus* in Liaoning and provide scientific support for the formulation of precise prevention and control strategies.

## 2 Materials and methods

### 2.1 Materials, reagents, and instruments

This study was approved by the Ethical Review Committee of Liaoning Provincial Center for Disease Control and Prevention, and all fecal sample collection was conducted with the informed consent of the patients or their legal representatives. Foodborne disease cases were defined as patients with diarrhea as the main symptom (≥3 bowel movements in 24 h) and abnormal stool characteristics (loose, watery, mucous, or bloody stools); cases with diarrhea caused by antibiotic use or improper chemical ingestion were excluded (12). Fecal samples from foodborne disease cases were collected from 2023 to 2024. Pathogen screening was conducted in hospital laboratories, and the final identification and confirmation were completed by Liaoning Provincial Center for Disease Control and Prevention. A total of 261 clinical *Vibrio parahaemolyticus* isolates were obtained in this study, and no food-derived isolates were included in the subsequent genomic sequencing and analysis.

SSNP-9600A Automatic Nucleic Acid Extractor (Jiangsu Shuoshi Biotechnology Co., Ltd.); IMH100-SSS Thermostatic Incubator (Thermo Fisher Technologies, Inc., USA); Milli-Q Pure Water Meter (Merck Millipore, Germany); VITEK2 automatic microbial biochemical identification system (BioMerieux, France).

Three percentage alkaline peptone water (APW), 3% tryptic soy agar (TSA) (Beijing Luqiao Technology Co., Ltd.), Vibrio chromogenic medium (Chromagar, France), and bacterial DNA extraction kit (Jiangsu Shuoshi Biotechnology Co., Ltd.). All reagents were quality-controlled and used within their expiration dates.

### 2.2 Strain identification

Magnetic beads containing *Vibrio parahaemolyticus* were enriched using 3% APW selective broth. The enriched broth was streaked onto *Vibrio* chromogenic agar plates for screening. Suspicious colonies were picked and inoculated onto 3% TSA plates for purification. Purified colonies were subjected to confirmatory testing using the automatic microbial biochemical identification system.

### 2.3 Nucleic acid extraction and whole-genome sequencing

Nucleic acid extractor was performed using the SSNP-9600A Automatic Nucleic Acid Extractor (Jiangsu Shuoshi Biotechnology Co., Ltd.). WGS was performed on the Illumina platform with paired-end (PE150) sequencing; the insert fragment size was set to be less than 400 bp, generating 150 bp single-end read lengths. The raw sequencing data were quality-controlled, and optimized clean reads were assembled using SPAdes (13), and the contigs were then mapped back to the clean reads for correction. Scaffolds were constructed by partial splicing and optimization of assembly results based on the overlapping and paired-end relationships of the reads. Genome assembly quality was evaluated using CheckM: the average sequencing depth was ≥100 × , the average N50 of the assembled genome was 3.26 Mb, the total assembly size was 4.8–9.3 Mb, the number of contigs was 2–28, the genome completeness was >95%. Only the assembled sequences meeting the above criteria were used for downstream analysis. Raw whole-genome sequencing reads of the 261 *Vibrio parahaemolyticus* isolates have been deposited in the NCBI under the BioProject accession number PRJNA1426136.

### 2.4 Multilocus sequence typing

To determine the strain's MLST type, sequencing results were assembled and then uploaded to the Center for Genomic Epidemiology's MLST analysis platform (https://cge.food.dtu.dk/services/MLST/). The sequences of seven alleles were used to identify the genotype (sequence type, ST). All inferences based on MLST results are limited to the resolution of MLST typing and do not involve evolutionary or transmission inferences beyond this genotyping method.

### 2.5 Virulence gene identification

To identify virulence genes, the sequenced genomes were compared to the virulence factor database (VFDB) (14). Gene coverage and sequence identity thresholds were set at 80% in the parameter settings, and annotations for virulence factor genes were acquired and statistically examined.

### 2.6 Identification of Antibiotic resistance genes

The strain's resistance genes were identified by comparing the genome sequence with ResFinder v4.0 (15) using ABRicate v0.5 (https://github.com/tseemann/abricate). In the parameter settings, 80% was chosen for both the gene coverage and sequence identity levels.

### 2.7 Data processing

The ST data of 261 *Vibrio parahaemolyticus* isolates were subjected to clustering analysis of MLST profiles using the unweighted pair group method with arithmetic mean (UPGMA) in BioNumerics 8.0 software, and minimum spanning tree (MST) and a similarity matrix were generated. Excel 2019 was used for data collation and sorting. SPSS 27.0 software was used for statistical analysis: χ^2^ tests were used to compare the detection rates of *Vibrio parahaemolyticus* between different years; False Discovery Rate (FDR) correction was applied for multiple comparisons in the statistical analysis of antibiotic resistance gene and virulence gene carriage rates across different epidemiological subgroups (e.g., age, occupation, eating venue, exposed food category) A difference with *P* < 0.05 was considered statistically significant. All heat map analyses were used to visually describe the descriptive association between genetic features and epidemiological characteristics; the color depth in the heat map represents the carriage rate percentage of the genes, with darker colors indicating a higher gene carriage rate percentage and lighter colors indicating a lower gene carriage rate percentage. All heat map analyses are exploratory in nature, without hypothesis-driven statistical texting and and causal inference. No multivariable regression modeling was performed due to the descriptive design of this surveillance study.

## 3 Results and analysis

### 3.1 Basic information

#### 3.1.1 Detection of *Vibrio parahaemolyticus*

A total of 10,413 foodborne disease cases were monitored in Liaoning province from 2023 to 2024, and 261 strains of *Vibrio parahaemolyticus* were identified, with an overall detection rate of 2.51%.

#### 3.1.2 Temporal distribution characteristics

The proportion and the positive rate of *Vibrio parahaemolyticus* in foodborne disease cases in 2024 were significantly higher than those in 2023, with a statistically significant difference in the detection rate between the two years (*P* < 0.05) (Table 1). Despite the significant difference in detection rates, 2023 and 2024 exhibited consistent seasonal epidemic dynamics of *Vibrio parahaemolyticus* infections: the positive *Vibrio parahaemolyticus* cases were predominantly concentrated in July–September in both years, with the highest number of cases in August, followed by July (Table 2). Positive cases were detected only in June, July, August, September, and October in two-year period, with the majority concentrated in July-September, indicating a stable seasonal epidemic pattern of *Vibrio parahaemolyticus* foodborne diseases in Liaoning province during 2023–2024.

**Table 1:** Distribution of *Vibrio parahaemolyticus* positive cases by detection year in Liaoning province, 2023–2024.

Year	Number of detected cases	Number of positive cases	Detection rate (%)	χ^2^ value	*P*-value
2023	4,864	62	1.27	53.97	< 0.05
2024	5,549	199	3.59		

**Table 2:** Monthly distribution of *Vibrio parahaemolyticus* positive cases in Liaoning province, 2023–2024.

Month	Number of cases	Number of positive cases	Detection rate (%)	χ^2^ value	*P*-value
January	98	0	0.00	194.25	< 0.05
February	175	0	0.00		
March	283	0	0.00		
April	373	0	0.00		
May	582	0	0.00		
June	1,037	1	0.10		
July	2,282	73	3.20		
August	2,503	137	5.47		
September	1,591	48	3.02		
October	688	2	0.29		
November	400	0	0.00		
December	401	0	0.00		

#### 3.1.3 Distribution of exposure foods

Epidemiological exposure survey showed that aquatic animals and their products were the primary exposure food category for *Vibrio parahaemolyticus* positive cases followed by various foods (23.75%) and meat and meat products (13.03%) (Table 3).This distribution pattern was consistent across 2023 and 2024 (exploratory observation without statistical validation).

**Table 3:** Distribution of exposure foods for *Vibrio parahaemolyticus* positive cases in Liaoning province, 2023–2024.

Exposed food category	Number of cases	Percentage
Aquatic animals and their products	136	52.11
Various foods	62	23.75
Meat and meat products	34	13.03
Fruit and fruit products	13	4.98
Grains and grain products	5	1.92
Beverages and frozen beverages	4	1.53
Vegetables and vegetable products	4	1.53
Dairy and dairy products	2	0.77
Fungi and fungal products	1	0.38

#### 3.1.4 Distribution of eating venues

According to the distribution of cases by dining venue, the majority of cases (37.55%) took place in restaurants, with homes coming in second (34.87%). See Table 4.

**Table 4:** Distribution of eating venues for *Vibrio parahaemolyticus* positive cases in Liaoning province, 2023–2024.

Place of consumption	Number of cases	Percentage (%)
Restaurant	98	37.55
Home	91	34.87
Food service industry–other	22	8.43
Street food	19	7.28
Food service industry	12	4.60
Other	9	3.45
Company cafeteria	4	1.53
Rural banquets	4	1.53
Food store	1	0.38
Food service–street food	1	0.3

### 3.2 Antibiotic resistance gene carriage status

Among the 261 *Vibrio parahaemolyticus* isolates, 94.63% (247/261) carried two antibiotic resistance genes, 3.8% (10/261) carried three genes, and 1.9% (5/261) carried 4–10 genes. *Tet*(34) and *bla*CARB-22 were the most prevalent resistance genes with carriage rates of 100% and 94.64%, respectively; other resistance genes, (e.g., *bla*CARB-31, *bla*CARB-25) had low carriage rates (< 5%). One isolates carried the maximum number of resistance genes (10 in total), including *bla*CARB-22, *bla*PER-1, *tet*(34), *sul*1, *sul*2, *qnr*VC6, *aac*6′)-Ib3, *cat*A3, *cat*B8, and *aad*A1.

Geographically, Dandong, Fushun, Shenyang, and Yingkou showed a consistent resistance gene carriage pattern, with 100% of isolates carrying *bla*CARB-22 and *tet* (34), and no other resistance genes detected. In contrast, Dalian, Huludao, and Anshan isolates all carried *tet* (34) (100%), and additionally harbored several other resistance genes at low levels: 18 resistance genes (e.g., *bla*CARB-18, *bla*CARB-31) were detected in Dalian isolates, and *bla*CARB-25, *bla*CARB-35, *dfr*A14, and *hug*A were detected in Huludao isolates, with *qnr*D1 showing a relatively high carriage rate (17.39%) in Huludao.

**Figure 1:**
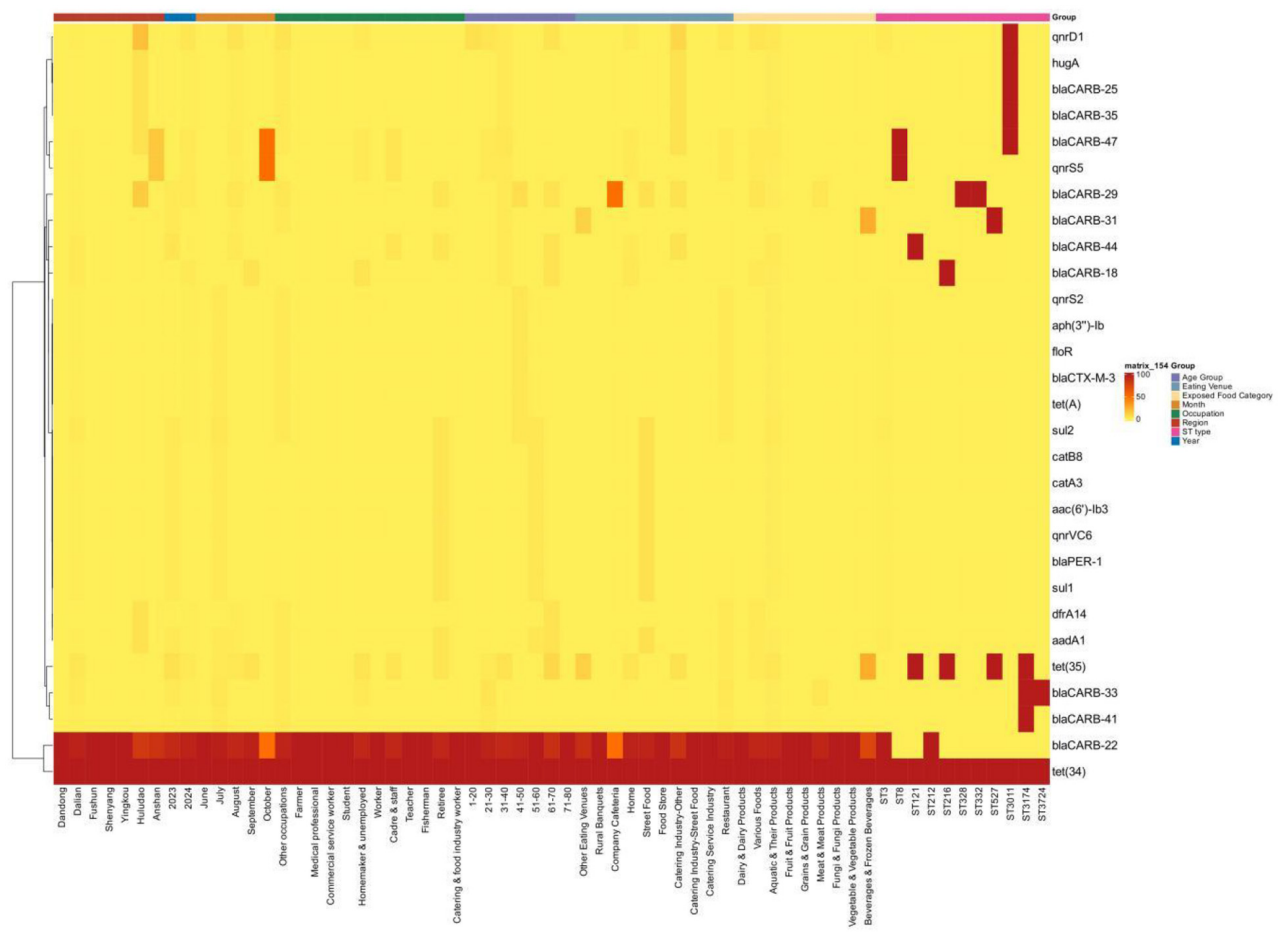
Heat map of different drug resistance genes and incidence area, incidence year, incidence month, patient gender, patient occupation, patient age range, eating place, exposed food category, and ST type. The heat map only presents descriptive associations between antibiotic resistance gene carriage and epidemiological factors, without causal inference. The color gradient represents the carriage rate (0–100%), with darker colors indicating higher carriage rates.

The distribution trend of antibiotic resistance genes was similar between 2023 and 2024, with slight monthly variations from June to September (descriptive observations without statistical validation). In October, the carriage rate of *bla*CARB-22 decreased to 50%, while that of *bla*CARB-47 and *qnr*S5 increased to 50%.B*la*CARB-22 was universally carried by isolates from most occupational groups (farmers, medical professionals, commercial service workers, students, workers, teachers, fishers, and food service workers), while retirees and other occupational groups carried a wider range of resistance genes (13 and 20 types, respectively). Resistance gene carriage showed little variation across different food exposure groups; *bla*CARB-22 was 100% present in isolates associated with dairy, fruits, grains, fungi, and vegetables, and 25% of isolates linked to beverages and frozen beverages carried *bla*CARB-31 and *tet* (35) (Figure 1).

### 3.3 Carriage of virulence genes

The number of virulence genes carried by the 261 *Vibrio parahaemolyticus* isolates ranged from 29 to 70. Among the 71 virulence genes detected 61 genes were identified as core virulence genes, carried by all isolates. The *tdh* gene, a major virulence marker encoding thermostable direct hemolysin, had a carriage rate of 96.17%. Ten genes showed lower carriage rates than others, such as *vsc*U2 and *flg*B. The *flg*B gene had the highest deletion rate (95.79%), followed by *vsc*U2 (83.91% absence).

Geographically, isolates from Shenyang, Yingkou, and Dandong showed high virulence gene carriage rates, with Shenyang and Yingkou isolates displaying similar carriage patterns; isolates from Anshan and Huludao showed variations in the frequencies of virulence gene carriage compared with other areas. The virulence gene distribution showed a stable trend between 2023 and 2024, with minor alterations in carriage rates from June to October, and no discernible differences between July and September (descriptive observations without statistical validation).

Regarding patient occupations, isolates from commercial service workers did not carry the *vsc*U2 and *flg*B genes, while 50% carried 18 virulence genes, including *vsc*Q2, *vcr*D2, and *tdh* (this is a descriptive association observed in the heat map and has not been statistically validated). Isolates from farmers, medical professionals, teachers, and food service workers carried all virulence genes except *vsc*U2 and *flg*B, with consistent carriage patterns among these occupational groups. Among all age groups, isolates frome 11 to 50 years old had the highest virulence gene carriage rates, while those above 51 years old showed a slight decrease.

**Figure 2:**
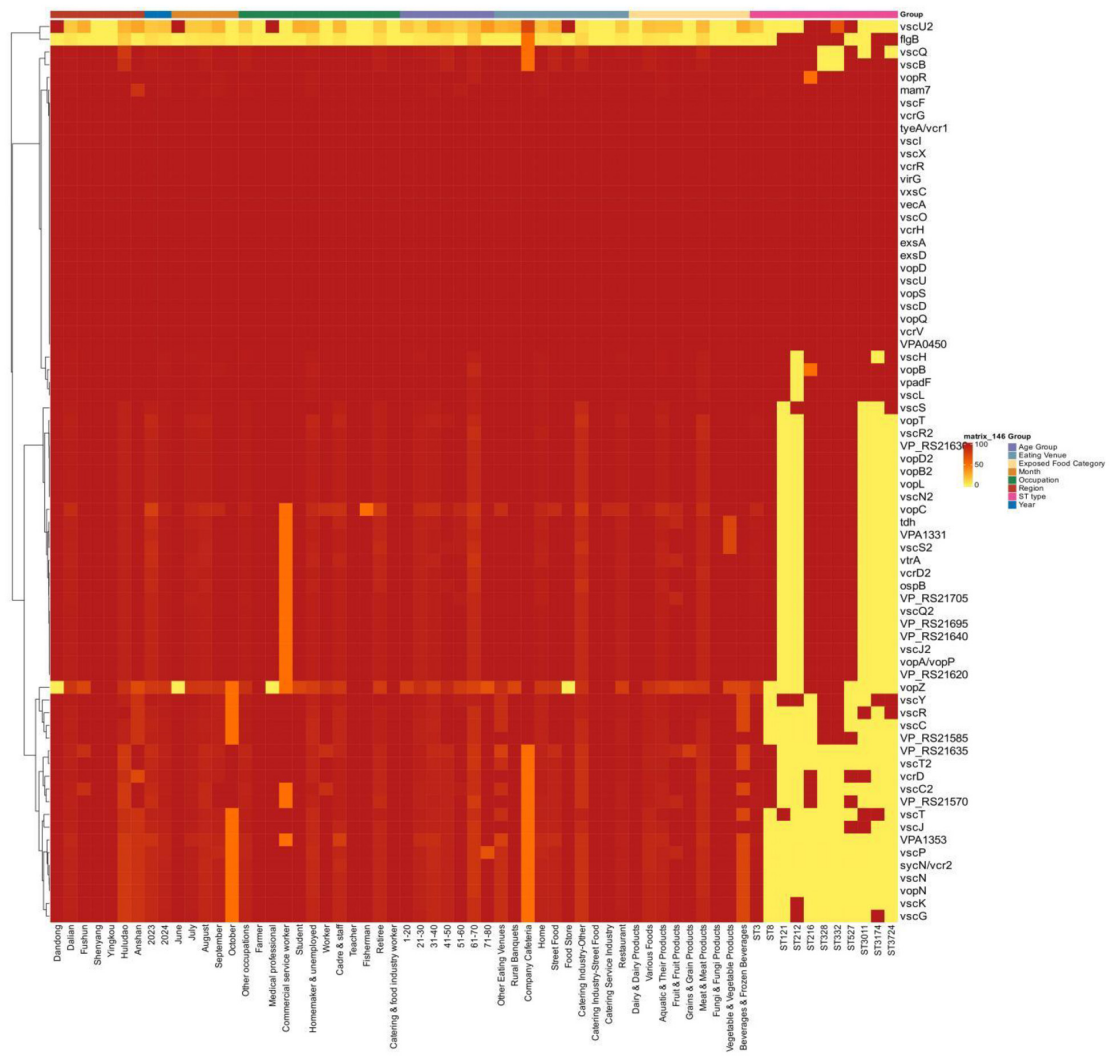
Heat map of different virulence genes and incidence area, incidence year, incidence month, patient gender, patient occupation, patient age range, eating place, exposed food category, and ST type.

In terms of exposed food, strains from dairy and fungi carried all virulence genes except *vsc*U2 and *flg*B with consistent patterns. Isolates from aquatic animals and their products carried virulence genes to varying degrees, with slightly lower carriage rates of *vsc*U2 and *flg*B: beverages and frozen beverages showed distinct virulence gene carriage patterns with lower carriage rates for some genes, while other food categories had relatively consistent carriage rates. Among dining settings, strains from restaurants and homes exhibited the highest virulence gene carriage, while those from company cafeterias, rural banquets, food stores, and street food showed relatively lower carriage. Higher virulence gene carriage in these strains is a potential genomic feature (presence only) and does not directly indicate a higher pathogenic potential, gene expression level or clinical severity of the infection (Figure 2).

### 3.4 MLST typing, virulence genes, antimicrobial resistance genes, and epidemiological characteristics of 261 vibrio parahaemolyticus isolates

Comparative analysis based on MLST typing revealed 11 ST types among the 261 *Vibrio parahaemolyticus* isolates, with ST3 as the absolute dominant type (94.25%, 246/261) and other ST types accounting for a small proportion.

ST3 was widely distributed in seven cities of Liaoning province, with the highest proportion in Dalian (84.55%), followed by Huludao (7.72%). Non-ST3 isolates were mainly concentrated in Dalian (eight ST types), with distinct genetic profiles based on MLST typing. ST3 was the dominant type in both 2023 and 2024 with a slight increase in 2024, and ST121 was only detected in 2023. ST3 showed a high prevalence in all months, with 100% in June and 97.26% in July; August had the most diverse ST types (eight types), which is a descriptive observaton of genotypic diversity.

**Figure 3:**
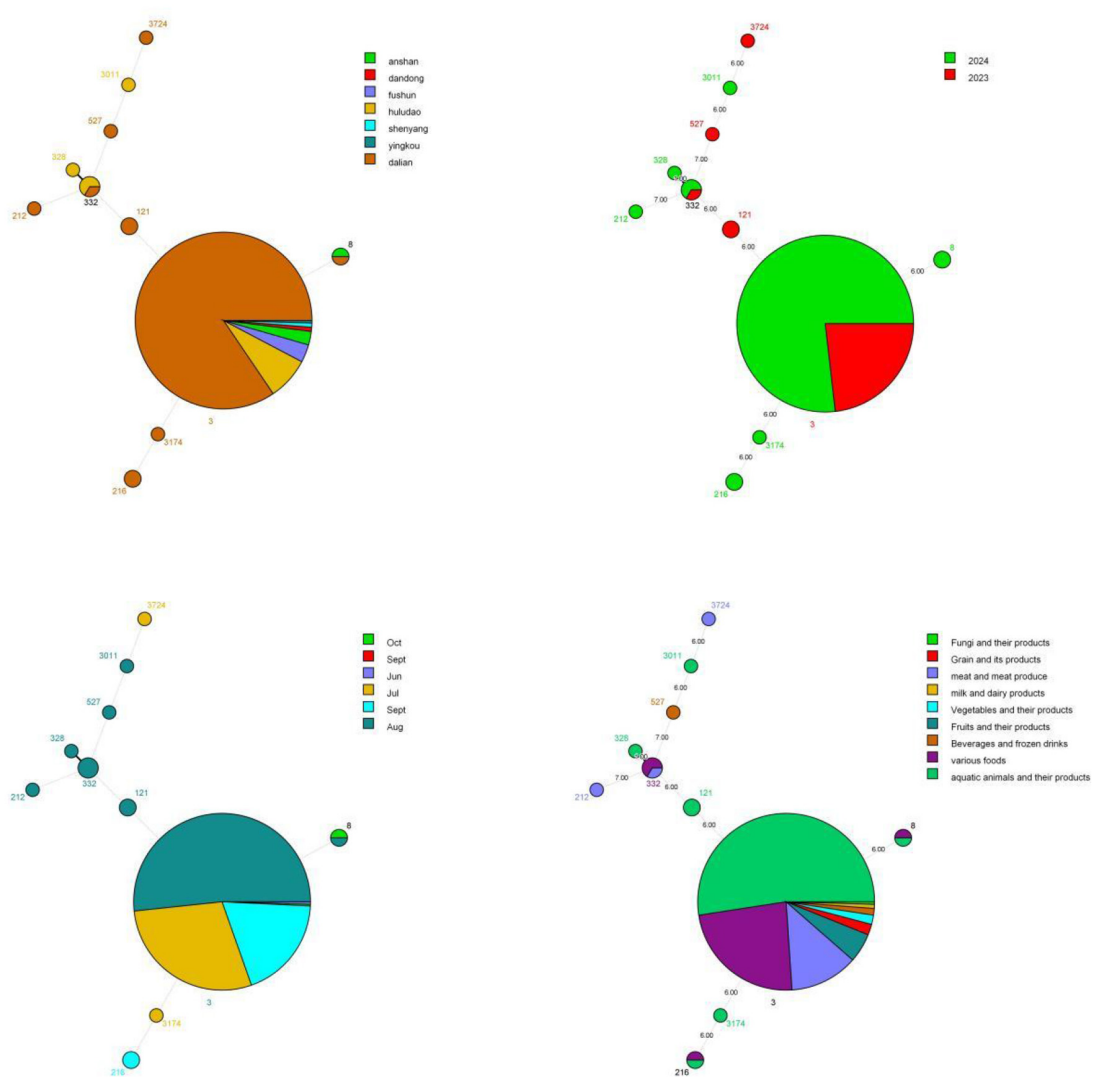
Minimum spanning tree (MST) analysis of 261 *Vibrio parahaemolyticus* isolates based on different epidemiological characteristics: MST of incidence area and ST type **(top left)**, MST of onset year and ST type **(top right)**, MST of onset month and ST type **(bottom left)**, MST of exposed food category and ST type **(bottom right)**.

ST3 was universally detected in isolates from farmers, medical professionals, students, and workers, and showed a stable high proportion across all age groups (100% in 11–20 and 51–60 age groups). In terms of food exposure groups, ST3 was 100% prevalent in isolates associated with dairy, fruits, grains, fungi, meat, and vegetables, while aquatic animals and their products showed higher ST genotypic diversity. For eating venues, ST3 accounted for 100% of isolates from rural banquets, food stores, food service-street food, and food service industries, and over 90% from homes, street food vendors, and restaurants. ST332 was the most prevalent non-ST3 type in company cafeterias (50%) (Figure 3).

Cluster analysis based on MLST allelic variation showed that the 11 ST types were clustered into 10 groups, with ST3174 and ST3724 showing close genetic similarity based on MLST typing. ST3 formed a distinct genetic cluster independent of other ST types based on MLST resolution, and exhibited a high virulence gene carriage rate (100% for 43 virulence genes and complete deletion of *flg*B). Non-ST3 isolates had 100% carriage rates for 21 virulence genes (e.g., *vcr*V, *vsc*D, and *vop*Q), with lower carriage rates for other virulence genes and complete deletion of *vsc*N, *vop*N, *syc*N/*vcr*2, *vsc*P, and VPA1353. No inferences about gene transfer are made from the co-occurrence of resistance genes across ST types, as this requires further genomic analysis.

Different ST types had distinct resistance gene profiles: ST3 carried *bla*CARB-22 at 100% and uniquely harbored *aad*A1, *aph*3”)-Ib, *bla*CTX-M-3, *bla*PER-1, *cat*A3, *cat*B8, *dfr*A14, *flo*R, *qnr*S2, *qnr*VC6, *sul*1, *sul*2, and *tet* (A) genes. ST216 uniquely carried *bla*CARB-18; ST527 uniquely carried *bla*CARB-25 and *bla*CARB-31; ST3011 uniquely carried *bla*CARB-35 and *hug*A gene, ST3174 exclusively carried *bla*CARB-41 gene, ST121 exclusively carried *bla*CARB-44 gene, and ST8 exclusively carried *qnr*S5 gene.

### 3.5 MLST typing, virulence genes, and epidemiological characteristics of isolates from patients with different symptoms

#### 3.5.1 Gastrointestinal symptoms

The virulence genes *vsc*F, *vxs*C, *vcr*V, and 17 other genes maintained a 100% carriage rate across all isolates from patients with gastrointestinal symptoms. In contrast, *vsc*U2, and *flg*B exhibited lower carriage rates (16.09% and 4.21%, respectively). No correlation was found between virulence gene distribution and the severity of gastrointestinal symptoms. In terms of ST types, ST3 was the most frequently detected ST type in isolates associated withgastrointestinal symptoms (82 symptoms), significantly more than other ST types. ST212, ST121, ST527, and other non-ST3 types were only linked to 1–2 gestrointestinal symptoms. Among all 87 gestrointestinal symptoms, 84 occurred in only 1–3 ST types, with just 3 symptoms appearing in more than 3 ST types.

#### 3.5.2 Systemic symptoms

Isolates carrying ≥68 virulence genes exhibited a higher proportion of systemic symptomatic cases, with fatigue, fever, and dehydration being the most common symptoms. Isolates carrying ≤ 67 virulence genes showed a higher proportion of asymptomatic infection, with pallor as sa characteristic sign (sample size was modest). Twenty virulence genes, including *vcr*V, VPA0450, *vop*Q, *vop*S, and *vsc*D showed a 100% carriage rate in all symptom categories (including asymptomatic isolates). Isolates from the fever (low-grade) symptoms group showed distinct virulence gene carriage patterns compared with other groups: genes such as *vcr*D, *vsc*C2, *vop*C, VPRS21635, *vsc*Q, *vsc*T2, and *vsc*B were not 100% present in this group, while these genes were universally carried in isolates from other symptom groups. Twenty-four virulence genes, including *vpad*F, *vcr*D2, and *mam*7, were 100% prevalent in all symptomatic groups but not in the asymptomatic group.

In terms of ST types, 93.69% of the asymptomatic group belonged to ST3. ST3 was most frequently detected ST type in all symptomatic groups except for “fever (low-grade)” and “pallor, dehydration, thirst, weight loss, fatigue”. 21.54% of ST3 strains were associated with systemic symptoms, while 78.46% were not. Two ST8 strains were detected: one was asymptomatic, while the other was associated with “pallor, dehydration, thirst, weight loss, fatigue”(100% incidence). Only ST332 exhibited a low-grade fever incidence of 33.33% among non-ST3 types; no other non-ST3 types were associated with systemic symptoms.

## 4 Discussion

Analysis of foodborne disease surveillance data from Liaoning Province during 2023–2024 showed that *Vibrio parahaemolyticus* positive cases clustered in July–September, with the highest number of cases recorded in August, and 2023 and 2024 exhibited consistent seasonal epidemic dynamics despite the significant increase in detection rate in 2024. This suggests that temperature may be a significant epidemiological determinant and confirms that *Vibrio parahaemolyticus* infections are more common in warmer months (16). This finding is consistent with other studies that have reported a higher risk ofpathogen contamination in seafood cold chain transport during summer heatwaves (17, 18). The favorable conditions for microbial growth in hot and humid environments likely explain why this seasonal distribution pattern is consistent with monitoring results from other parts of China. Additionally, epidemiological exposure survey statistics show that the most commonly implicated foods are high-protein, perishable categories such as meat and meat products, as well as aquatic products and their derivatives. This aligns with the pollution patterns revealed by historical monitoring data from Liaoning Province and monitoring results from other regions.(19, 20). Aquatic environments and seafood products, especially shellfish, crabs, and their processed products, are the primary habitats of *Vibrio parahaemolyticus* (21). The risk of *Vibrio parahaemolyticus* contamination in these products is significantly increased by improper handling during harvesting, processing, transportation, and sale (22). This inference of aquatic products as the main exposure source is based solely on epidemiological data, without genomic comparison evidence between clinical and food-derived isolates.

Of the 71 virulence genes identified in this investigation, 61 constitute the core virulence genome that all strains share. The thermostable direct hemolysin is encoded by the *tdh* gene, which has a 96.17% carriage rate. The heat-stable direct hemolysin protein forms a polar tetrameric structure that binds to eukaryotic cell membranes, causing transmembrane ion imbalance and is considered a key virulence-associated factor linked to diarrheal symptoms. The 96.17% *tdh* gene carriage rate in this study aligns with the 90% *tdh* presence in clinical isolates of *Vibrio parahaemolyticus* (19). Among the 71 virulence genes, 10 genes show different degrees of deletion, which may represent the genotypic differences between strains (no inference of pathogenicity). *Flg*B is only present in 11 strains, with a deletion rate of 95.79%, suggesting that the gene may be non-essential in most strains based on genomic presence. Twenty virulence genes, such as *vsc*F, *vxs*C, and *vcr*V, maintained a 100% frequency across all gastrointestinal symptoms in relation to gastrointestinal symptoms, suggesting that these core virulence genes may be essential genotypic components associated with gastrointestinal infection (no causal inference). No clear pattern was observed between the distribution of virulence genes and the severity of gastrointestinal symptoms, nor was there any correlation between virulence genes and symptom severity. Further studies are needed to elucidate their specific roles in gastrointestinal pathogenesis, including gene expression and phenotypic validation. To improve statistical power, we also recommend increasing the sample size, particularly for cass with severe symptoms. For systemic symptoms, genes including *vcr*V, VPA0450, *vop*Q, *vop*S, and *vsc*D exhibited 100% carriage rates across all systemic symptom groups, including asymptomatic isolates. Clinical symptoms did not affect their presence, indicating that these genes might be essential genotypic components intimately linked to the basic survival strategies of *Vibrio parahaemolyticus*. The molecular mechanisms behind these genotypic differentially should be examined in future research using larger sample sizes and functional validation experiments.

*Tet* (34) and *bla*CARB-22 were the dominant resistance genes with high carriage rates, which is consistent with the previous report of *bla*CARB gene carriage in *Vibrio parahaemolyticus* isolates from aquaculture in Liaoning province (19). However, in this study, only the carriage of resistance genes was detected by WGS, without *in vitro* phenotypic antimicrobial susceptibility testing, so the actual drug resistance of the isolates cannot be determined, and the clinical significance of *tet* (34) and *bla*CARB-22 needs to be further verified by combined genotypic and phenotypic analysis. Ten resistance genes involving antibiotics from the following classes—tetracyclines, sulfonamides, quinolones, aminoglycosides, chloramphenicol, and β-lactams—were present in one of the 261 strains. This strain spanned seven categories of resistance genes and carried considerably more resistance genes than typical strain, and its epidemiological transmission mechanism warrants monitoring. Aquatic animals and their products are the food category associated with this isolate. Notably, the prolonged and extensive use of antibiotics in modern aquaculture may exert significant selective pressure. Persistent exposure to antibiotics at subinhibitory concentrations in aquaculture environments may contribute to the dissemination and inheritance of these resistance genes in *Vibrio parahaemolyticus* populations, and further functional studies are needed to verify this hypothesis (23, 24).

Eleven different ST types were identified by multilocus sequence typing of 261 *Vibrio parahaemolyticus* isolates; ST3 was the most common type (94.25%), suggesting that it is the main epidemic strain in Liaoning province based on MLSTgenotyping, consistent with the dominant ST types observed in other regions of China (25). Very small percentages of all isolates were of other ST types, suggesting other infection origins or imported cases (descriptive inference only). Among different regions within Liaoning Province, Dalian isolates exhibited the highest number of ST types, encompassing nine ST types. ST3 was predominant, while the remaining eight ST types showed distinct genotypic profiles based on MLST typing. The primary food category implicated in Dalian isolates was seafood, indicating complex sourcing of seafood products in the city. The majority of the strains from the other six cities were ST3, which may share genotypic similarity with Dalian isolates based on MLST. Huludao's isolated ST types, on the other hand, showed distinct genotypic profiles from ST3 but shared a cluster with Dalian ST types based on MLST, indicating potential epidemiological connections (descriptive observation only). Consequently, source investigations for non-ST3 strains and market surveillance in Dalian should be prioritized. Between 2023 and 2024, the distribution of ST types stayed consistent, with ST3 showing exceptionally high prevalence in both years, indicating its continued dominance as the circulating strain in the two-year period. Only in 2023 did ST121 emerge, and it may have been subsequently controlled. August produced the most isolates and the greatest number of ST types (eight), suggesting complex sources of infection. August appears particularly conducive to *Vibrio parahaemolyticus* circulation and transmission, with infections arising from diverse genotypic strains. The prevalence of seafood as the primary exposure source in August further suggests multiple seafood sources entering the market during this month. In contrast, isolates from July, September, and October showed more concentrated genotypic sources, with minimal variation, suggesting fewer distinct strains entering the market. Among different occupations, farmers, medical personnel, students, and workers all exhibited 100% ST3 prevalence, although these groups had relatively fewer reported cases, highlighting the universal exposure to the ST3 epidemic strain. ST3 was the predominant ST type across all food categories. Aquatic products and meat products exhibited higher ST genotypic diversity with greater genetic differences based on MLST, suggesting distinct supply chains. Enhanced oversight of externally sourced foods is warranted.

The 11 ST types are grouped into 10 different clusters according to the minimal spanning tree diagram (based on MLST allelic variation). ST3 exhibits unique characteristics, clearly distinct from all other types based on MLST resolution, indicating its distinct epidemiological profile. It demonstrates high carriage rates of virulence genes (100% for 43 virulence genes, genomic presence only), a genotypic feature associated with its epidemic dominance. Non-ST3 strains exhibit lower carriage rates for most virulence genes compared to ST3, except for 21 genes, which are 100% present, suggesting genotypic differences in virulence gene profiles between ST types (no inference of pathogenic superiority). This ST-specific genotypic pattern may imply genotypic divergence among these strains. Subsequent studies will investigate associations between specific ST types and virulence gene deletions, employing whole-genome sequencing to decipher virulence island-level transfer pathways. This research will provide crucial insights into pathogenic mechanisms and control strategies, laying a scientific foundation for implementing precision molecular epidemiological prevention and control. Regarding resistance genes, *bla*CARB-22 was dected in 100% of ST3 an ST212 isolates; further genomic analyses are required to investigate potential horizontal gene transfer of this gene between different ST types, as co-occurrence alone does not provide evidence for gene transfer. Each ST type carries distinct sets of resistance genes, indicating a degree of type-specific genotypic profiles for antibiotic resistance. Regarding systemic symptoms, ST3 was the most frequently detected ST type in all symptomatic groups except “fever (low-grade)” and “pallor, dehydration, thirst, weight loss, fatigue.” Other ST types showed low prevalence for most symptoms, a finding based on small sample sizes and exploratory observation only. In terms of gastrointestinal symptoms, ST3 was the most prevalent ST type associated with all gastrointestinal symptoms, and was linked to 82 different symptoms (significantly more than other ST types), which is consitent with its high virulence gene carriage profile. Prioritized surveillance for ST3 is justified because other ST types displayed fewer associations with symptoms. A major limitation of this study is the exclusive inclusion of clinical isolates without the collection and genomic analysis of food-derived isolates, which prevents the direct verification of the foodborne transmission of *Vibrio parahaemolyticus* and the identification of the exact contamination source of clinical strains at the genomic level. Future research will include paired sequencing of clinical and food-derived isolates to clarify the molecular transmission chain between food and humans.In addition, this study only detected the genomic presence of virulence genes and antibiotic resistance genes, without phenotypic validation, which limits the inference of actual pathogenicity and drug resistance of the isolates. Further functional studies combining genotyping and phenotyping are needed to verify the clinical significance of the detected genes. All subgroup analyses in this study are exploratory and descriptive, without multivariable regression modeling; future studies with a larger sample size and hypothesis-driven design are needed to confirm the associations between genetic profiles and epidemiological characteristics.

## Data Availability

The raw whole-genome sequencing reads of the Vibrio parahaemolyticus isolates generated and analysed in the present study are publicly available in the National Center for Biotechnology Information (NCBI) Sequence Read Archive (SRA) under BioProject accession number PRJNA1426136 (https://www.ncbi.nlm.nih.gov/bioproject/PRJNA1426136/). All other relevant data supporting the conclusions of this study are included within the article. Further enquiries regarding the data may be directed to the corresponding authors (Wenli Diao, diaodwl@163.com; Yan Wang, wy-0124@163.com).
